# Virtual Therapeutic Garden: A Promising Method Supporting the Treatment of Depressive Symptoms in Late-Life: A Randomized Pilot Study

**DOI:** 10.3390/jcm10091942

**Published:** 2021-05-01

**Authors:** Joanna Szczepańska-Gieracha, Błażej Cieślik, Anna Serweta, Krzysztof Klajs

**Affiliations:** 1 Faculty of Physiotherapy, University School of Physical Education in Wroclaw, 51-612 Wroclaw, Poland; joanna.szczepanska@awf.wroc.pl; 2 Faculty of Health Sciences, Jan Dlugosz University in Czestochowa, 42-200 Czestochowa, Poland; 3 Department of Physical Education, University School of Physical Education in Wroclaw, 51-612 Wroclaw, Poland; serwetanna@gmail.com; 4 Polish Milton H. Erickson Institute, 94-036 Lodz, Poland; krzysztof.klajs@gmail.com

**Keywords:** virtual reality, head-mounted display, total immersion, elderly, mental health, Ericksonian psychotherapy, depression, stress

## Abstract

The multifactorial genesis of old-age depression requires multi-professional therapy combining physical activity and psychosocial interventions; however, there is still a percentage of older people who do not exhibit satisfactory improvements. The aim of this study was to evaluate the effectiveness of virtual therapy in the elderly for whom the previous multimodal, biopsychosocial therapeutic programme had not brought the expected results. Twenty-five elderly women with depressive symptoms were randomly divided into a virtual reality group (VR, *n* = 13) and a control group (Control, *n* = 12). The average age was 70.73 and the average intensity of depression symptoms amounted to 12.26 in the Geriatric Depression Scale (GDS-30). As a virtual reality source, the VRTierOne (Stolgraf^®^) device was used. The therapeutic cycle consisted of eight virtual therapy sessions, twice a week for four weeks. As primary and secondary outcome measures, the GDS-30 was performed at three time points. In the VR group, the GDS-30 score was reduced by 36%, and the result persisted in the follow-up tests. Immersive virtual therapy significantly lowered the intensity of depressive symptoms, as well as stress and anxiety levels in older women taking part in the group-based multimodal therapeutic programme, whose earlier therapy had not brought the expected results.

## 1. Introduction

Depressive disorders in the elderly are common and can manifest either at a younger age and reoccur later in life, or have an onset at above 60 years of age. On the basis of a literature review covering 24 international community-based elderly population studies, the prevalence of major depression ranged from 4.6% to 9.3%, and that of depressive disorders from 4.5% to 37.4% [1]. A special prevalence rate of depressive disorders in nursing home residents was reported by Anstey et al. For women, rates range from 8.4% to 61.4%, and from 6.4% to 38.1% for men [2]. Disability, especially in self-care or social activities, is strongly related to late-life depression [3]. Women scored higher on the affective suffering factor [1].

The studies published in The Lancet Psychiatry in 2018 showed that older patients have a worse prognostic course of major depressive disorders over a two-year follow-up period than younger individuals [4]. The elderly, when compared to younger patients, were found to be more likely to show a persistent depression diagnosis, unsatisfactory improvements in depressive symptoms and were less likely to remit. Late-life depression has a higher tendency to become chronic and implies an increased risk of progression [4]. The multifactorial genesis of depression in old age includes psychosocial, vascular and metabolic factors and requires multimodal and multi-professional therapy combining physical activity with psychosocial interventions [5]. Such a model is based on the biopsychosocial approach to treating depression [6].

In recent years, there has been a growing interest in the use of modern technologies in the treatment of mental disorders. Growing social needs and the lack of an adequate number of qualified specialists are determining new directions of research. In the meta-analysis published in Nature in 2018, the authors concluded that VR treatment had moderate to large effects compared to the control conditions, making it an additional effective choice available to clinicians and patients [7]. The cited meta-analysis takes into consideration depressive or anxious symptomatology but in patients suffering from anxiety (flight anxiety, panic disorder, post-traumatic stress disorder, social anxiety). Therefore, the results of the present experiment, despite the small size of the study group, are an important addition to existing knowledge. Moreover, the review on the use of virtual reality in psychiatric disorders and rehabilitation [8,9,10,11] carried out by our team has demonstrated that there is a gap of scientific reports related to the possibility of using VR therapy, especially based on total immersion, in older patients with depression.

The additional, very interesting aspect of the therapeutic solution analysed by our team refers to the use of clinical hypnosis in order to broaden the scope of therapeutic methods. In the earlier years, it was found that VR may have the utility as an effective medium for hypnosis [12,13] and it is an effective intervention in lowering the levels of pain and anxiety in patients with burn pain [14], decreasing the stress level [15], or treating Post Traumatic Stress Disorder (PTSD) [16]. The results presented by Thompson et al. seem to be extremely interesting; they found on the basis of conducted Randomized Controlled Trial (RCT) that for individuals with low levels of trait absorption, self-rated psychological engagement (vividness of imagery, freedom from distraction, sustained concentration) was significantly higher when hypnosis with VR imagery was used. Moreover, the authors reported that the wearing of the VR head-mounted display and maintaining eyes open did not appear to inhibit hypnotic relaxation [17]. In the study being described, there were only young subjects (psychology students), whereas in our study we included only the elderly.

The developed therapy is the first attempt to transfer the suppositions of Ericksonian psychotherapy to the VR environment. The basic assumption in this therapy is that in order to achieve improvement in the mental state of the patient, you do not have to talk to him or her directly about their problem. Instead, you can create a situation that metaphorically reflects the essence of their problem and shows possible solutions [18,19]. This approach is fundamentally different from cognitive-behavioural therapy, which is more often transferred to a virtual environment. The most important therapeutic tool in prepared VR therapy is telling a story that metaphorically reflects the patient’s situation and helps to find a good solution to a problem or a direction for personal development. Hypnotic suggestions help enhance the belief that positive changes in a patient’s life have begun and will be sustained in the future [20,21,22,23]. This is in line with the research trend proposed almost 10 years ago, referred to as Positive Technology [24,25]. It aims to combine elements of positive psychology and new technologies and tries to answer the question of how digital technologies could help shaping positive human functioning [26].

The aim of the present study was to assess the effectiveness of virtual therapy based on the idea of a Virtual Therapeutic Garden, applied to older people in whom the previous group-based multimodal therapeutic programme did not bring the expected effects.

## 2. Materials and Methods

### 2.1. Overview

The study was conducted in the Foundation for Senior Citizen Activation, “Siwy Dym”, in Wroclaw, Poland. The facility runs a therapeutic programme for older women, whose family physician determines the severity of depressive symptoms 10 or more on the Geriatric Depression Scale (GDS-30).

However, around 30% of the subjects did not show the expected improvement and required individual therapeutic support [5]. The study included people who, despite regularly participating in the therapeutic programme for at least three months, did not experience a satisfactory reduction in depression symptoms and anxiety levels. All the subjects gave their written consent to take part in the project. The study received the consent of the Ethical Committee of the University School of Physical Education in Wroclaw, Poland (No. 32/2019, applied: 17 May 2019; received: 31 May 2019).

The study was designed as a parallel-group RCT with a masked outcome assessor and with measures repeated across three-time points: pre intervention, post intervention and 2 weeks after the intervention. There was no sample size calculation performed. To enroll participants, we applied the rule of thumb from the recommendations for a pilot study by Julious (2005) and Whitehead et al. (2015) [27,28]. The design follows the recommendations for the second phase (VR2) of clinical trials in health using virtual reality, focusing on acceptability, feasibility, tolerability and initial clinical efficacy [29]. The protocol was registered in the government ClinicalTrials.gov PRS database after the participant recruitment began (registration number: NCT04047511). The reason for the delay was related to the delayed acceptance of the PRS account by the University. The authors confirm that all ongoing and related trials for this intervention have been registered. Participant recruitment started on 01 June 2019 and follow-up was completed on 31 August 2019.

### 2.2. Participants

**Figure 1:**
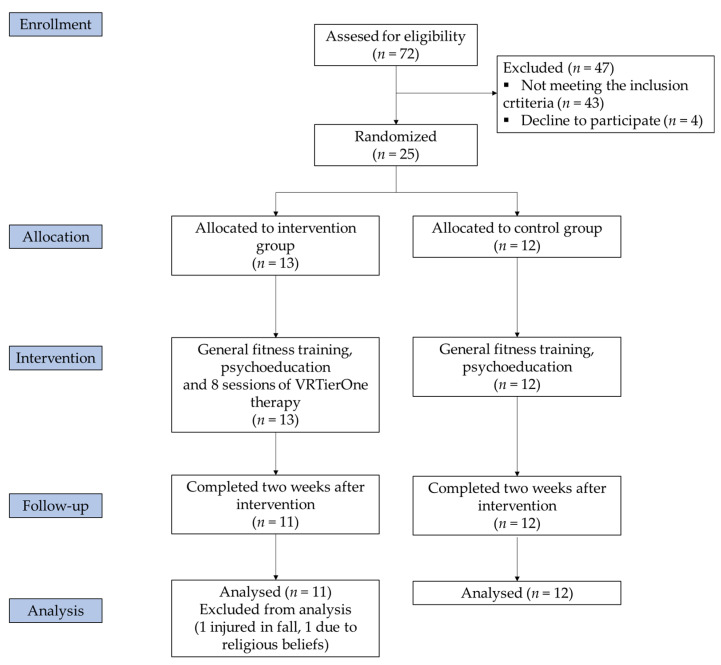
Study flow diagram.

Out of 72 women participating in a multimodal therapeutic programme, 29 did not achieve the expected improvement in depressive symptoms after three months of participation in programme, and, therefore, were enrolled for the study. However, four of them declined to participate in the study. Finally, 25 were recruited for this study. The subjects were randomly (coin flip) divided into two groups: the VR group (*n* = 13) and the control group (*n* = 12). The control group received the standard treatment (40 min in general fitness training and 20 min of health-promoting education and psychoeducation twice a week), while the VR group received the same treatment plus virtual reality therapy. The group assignment took place before the intervention. Two individuals were excluded from the study (one due to her religious beliefs, and one injured in an accident not connected with the project). Finally, for the statistical analysis, 11 participants were qualified for the VR group and 12 for the control group. Figure 1 describes the participants’ enrolment procedure. The inclusion criteria were as follows: age above 60, GDS score ≥ 10, or HADS-A ≥ 8, or HADS-D ≥ 8. The exclusion criteria included the presence of cognitive disorders, substance abuse preventing participation in the project, ongoing pharmacological treatment for depression, contraindications for virtual therapy (epilepsy, eye problems, labyrinth disorders) or participation in another therapeutic project or individual psychotherapy.

### 2.3. Outcome Measures

As a primary outcome measure, the 30-item Geriatric Depression Scale (GDS-30) was used. It is a self-rating screening tool to measure depression symptoms in older adults. It contains 30 “yes” or “no” items. A result between 0–10 points indicates a lack of depression, while a score of 11 or above indicates depression of increasing severity. The scale provides a high reliability (Cronbach’s *α* = 0.69–0.99) and validity [30]. 

As secondary outcome measures, the Perception of Stress Questionnaire (PSQ) and Hospital Anxiety and Depression Scale (HADS) were used. The PSQ was created by Plopa and Makarowski [31]. It is a 27-item scale scoring from 1 to 5 points for each item, where 21 items examine the level of stress in the areas of emotional tension, external stress and intrapsychic stress, and six items refer to the lie scale. The global scoring for the perception of stress ranges from 21 to 105, with a cut-off point of 60 for an elevated level of perceived stress. The higher the score, the greater the sense of stress. Cronbach’s *α* for the individual scale ranges from 0.69 to 0.80.

The HADS is a 14-item scale scoring from 0 to 3 points for each item. Seven items relate to anxiety (HADS-A), while the remaining seven relate to depression (HADS-D). The global scoring ranges from 0 to 42, with a cut-off point of 8/21 for anxiety and 8/21 for depression. The higher the score, the greater the level of anxiety or depression symptoms [32]. Cronbach’s *α* ranges from 0.78 to 0.93 for the HADS-A and from 0.82 to 0.90 for the HADS-D; test-retest correlation is *r* = 0.80 [33].

GDS-30 was selected as the primary outcome measure, and HADS as a secondary outcome measure because GDS-30 has much higher responsiveness to change than HADS-D. This could be due to several issues. First, the GDS-30 has 30 items on depression, and the HADS-D only 7 items on it. Smarr and Keefer (2011) published a study comparing the most popular measures of depression and depressive symptoms [34] which concluded that the construction of the HADS-D minimises the effect of somatic disorders associated with depression. Moreover, according to the authors, HADS-D is designed to identify probable “cases” of anxiety or depression, but it is not a very good diagnostic tool [34]. For the above reasons, we have decided to base first of all on depressive symptoms detected by GDS-30, adding HADS scores as supplementary assessment.

The participants outcome measures were evaluated at three-time points: at baseline, after the intervention (week 4) and at a two-week follow-up (week 6).

### 2.4. Interventions

Participants of both groups attended support group meetings (twice a week, 60 min each time). Within these meetings, they took part in general fitness training (40 min), relaxation exercises, as well as in health-promoting education and psychoeducation (20 min). Each class was conducted by a physiotherapist and a psychotherapist in a small, fixed group of 10–12 women. The assumptions of the described model are in accordance with the newest recommendations and its effectiveness has been proven [5].

The VR group had an additional intervention using virtual reality. As a virtual reality source, the VRTierOne (Stolgraf^®^, Stanowice, Poland) device was used. The hardware consists of VR HTC VIVE goggles (2017) and two controllers (manipulators) plugged into a PC. Thanks to using a head-mounted display and the phenomenon of total immersion, VR therapy provides intense visual, auditory and kinaesthetic stimulation. It can have a calming and mood-improving effect, as well as help the patients recognise their psychological resources and motivate them to take part in the rehabilitation process.

**Figure 2:**
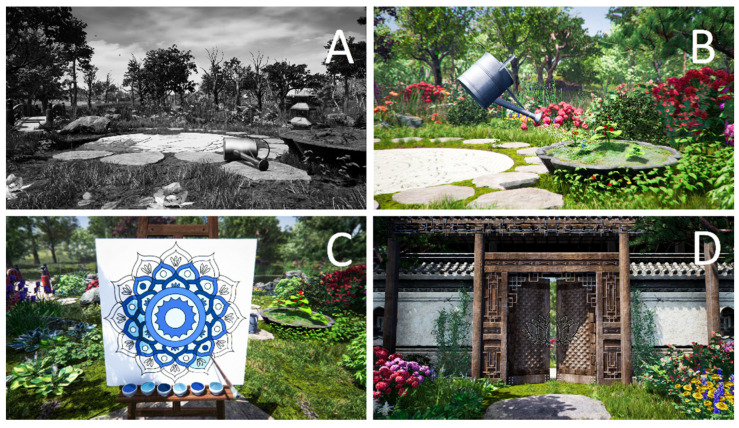
Garden of Revival screen captures: (**A**) initial stage of the therapy; (**B**) final stage of the therapy; (**C**) mandala colouring task; (**D**) gate leading to the Garden of Revival.

In the Virtual Therapeutic Garden, there is a rich set of symbols and metaphors based on the Ericksonian psychotherapy approach. The most important is the Garden of Revival, which symbolises the patient’s health. The metaphor of the garden, weakened and grey in the beginning (Figure 2A), and becoming more and more colourful and lively with every session symbolises the process of regaining energy and vigour (Figure 2B).

Thanks to the use of the controllers enabling interaction with the virtual world, the patient becomes an active participant in the therapy. During each session, in the central place of the virtual garden appears a mandala which symbolises various traits and emotions crucial to the therapy process, such as vitality, joy, optimism, diligence, creativity, inner wisdom and trust (Figure 2C). Emotions associated with specific colours and specially selected music become an illustration of subsequent sessions, accompanying the patient as he or she is immersed deeper and deeper in the virtual world.

The patient’s task is to colour the mandala with appropriate colours. Every task that the patient completes is rewarded by beautiful flowers appearing in the garden. The interaction between the user and the VR world intensifies the psychological immersion in the therapeutic story, thus strengthening the power of the metaphorical message. If any of the tasks prove too difficult, the computer instantly adjusts their level to the patient’s cognitive and kinesthetic abilities, so that he or she has to put effort into the task, but that it does not exceed his or her current capabilities. In this way, each session gives the patient the chance to succeed and obtain an appropriate emotional gratification, which motivates him or her to continue the therapy. At the end of the therapy, the visual effect of a beautiful, colourful garden is complemented with lively music and the sounds of nature (birds singing, water flowing, wind blowing).

The developed therapy has an original character and is based on many years of therapeutic practice and the following assumption: the sooner the patients understand or rather feel that they are responsible for their future and that there is a lot that they can do for themselves, the better the chance of regaining their physical and mental health. This is a process of transformation, where the patient—a person who is being treated/rehabilitated—becomes the person who feels co-responsible for his or her recovery process and its final results. The assumption is confirmed by research conducted in the neurological rehabilitation department, where 99 disabled patients were monitored [35].

This metaphorical and symbolic communication, relating primarily to the emotional processes is associated with the activity of the brain’s right hemisphere [36,37]. Each element of the virtual world has a certain symbolic function. However, the strength of Erickson’s psychotherapy lies in the fact that it is the patient who decides what meaning he or she will assign to the individual elements of this intricate world [19]. Therefore, the users should not be informed as to the specific meaning of each symbol. The more the patients tap into their inner wisdom, the greater the chance that they will assign to the perceived elements such meanings that they need most at the moment at hand [22,38]. The gate leading to the Garden of Revival (Figure 2D) is a gateway to unconscious psychological processes and resources, extremely important in the process of recovery [39].

To enhance the therapeutic effect, every session is enriched with breathing exercises (having a calming and relaxing effect and allowing better contact with the body), elements of the mindfulness training (for better concentration and intensification of immersion in the virtual world) and hypnotic suggestions (to enhance the belief that positive changes in patient’s life have just begun and they will be sustained in the future) [18,20,23]. These exercises and suggestions are incorporated into virtual therapy both before and after the mandala colouring task in such a way that they form a coherent whole. The content of the therapy was developed by a certified European Association of Psychotherapy therapist, who not only supervised the creation of the virtual therapy but also lent her own voice to the project, to make it sound as natural and reliable as possible. The project was also supervised throughout by the Director of Polish Milton Erickson Institute, who is a professional supervisor and chairman of the Scientific Department of Psychotherapy of the Polish Psychiatric Association. VR TierOne was produced under a grant from the Polish National Center for Research and Development. The same device has been used in recently published studies on cardiological and pulmonological rehabilitation [40,41].

In our study, there were eight sessions of VR therapy, each of them 20 min long. VR therapy was conducted twice a week for a period of four weeks.

### 2.5. Data Analysis

Statistica 12 (StatSoft) statistical software was used to perform all calculations and analyses. For continuous variables, the mean and standard deviations were reported. Percentages were used for categorical variables. The normality of the data was assessed by the Shapiro-Wilk test. The between-group differences in categorical data were compared using Chi-square tests, and continuous variables with independent *t* tests. One-way Repeated Measures Analyses of Variance (ANOVA) with a group as a categorical factor were applied, followed by the Bonferroni post hoc test. Mauchly’s test indicated that the assumption of sphericity had not been violated for any variable tested. The level of significance was set at *α* < 0.05.

## 3. Results

Out of 25 enrolled participants, 23 women completed the study. As presented in Table 1, no difference that would be statistically significant was observed between the study and control groups at baseline characteristics. While analysing the primary outcome, one can see the lack of differences between the groups in the pre-intervention study. In the VR group, after eight therapeutic sessions, there was a significant decrease in the GDS score (*F*(2,20) = 17.36, *p* < 0.001) by approximately 36% (12.27 vs. 8.27, *p* = 0.001) in the post-intervention tests, and the result persisted in the tests conducted two weeks after the intervention (8.27 vs. 7.27, *p* = 0.88). Over the same period, the GDS score did not change significantly in the control group (*p* = 0.61; Table 2 and Table 3).

## 4. Discussion

The present study was designed to assess the effectiveness of virtual therapy in older people whose previous group-based multimodal therapeutic programme did not achieve the expected results. Even though such a therapeutic model meets the requirements of the newest recommendations and its effectiveness has been empirically proven [5,6], there are individuals who additionally need individual therapeutic support. VR therapy and telemental health service seems to be particularly promising fields. In one of the first systematic reviews of game-based digital interventions for depression, published in 2014, nineteen research projects were subjected to a thorough analysis [42]. Only three of those experiments regarded older people [43,44,45]. Four types of game interventions were identified: psychoeducation and training, virtual reality exposure therapy, exercising, and entertainment. The authors prepared a meta-analysis on the basis of eight RCTs. Only two papers were related to older people [43,45]. The meta-analysis revealed a moderate effect of the game interventions for depression therapy in the post-treatment (*d* = −0.47, 95% CI −0.69 to −0.24). In the conclusions, the authors emphasised that the literature review and meta-analysis confirm the effectiveness of game-based digital interventions for depression, but more large-scale, high-quality RCTs with sufficient long-term data for treatment evaluation are urgently needed [42].

There have been an increasingly large number of studies conducted since then. In a review on virtual reality interventions for anxiety and depression published in 2018, 32 relevant articles were identified, but only five met the criteria for inclusion in systematic literature reviews, with the general number of participants receiving VR treatment equal to 88 [46]. Only two of them were RCTs and involved older people [47,48]. In the summary of the literature review, similarly to the paper from 2014, the authors recapitulate that the findings favoured VR treatment in alleviating anxiety and depression symptomatology. However, the existing evidence is insufficient in supporting the advantages of VR interventions as a standalone treatment over traditional therapy because of small sample sizes and a lack of high-quality research designs in clinical settings [46].

The comparison of the results of our experiment alongside the aforementioned RCTs with the participation of older people [43,45,47,48] is rather difficult. One of the papers refers to men only, war veterans with PTSD syndrome [43]. Beck Depression Inventory (BDI), which was used as an assessment method for depression disorders, makes it difficult to directly compare the results. Nevertheless, similarly to our study, the intensity of depression symptoms decreased significantly in the VR group; BDI total scores decreased by around 40% from moderate depression to mild depression (*p* = 0.003). In three other RCTs, Nintendo Wii Fits or KINECT Xbox 360s [45,47,48] were used. These popular exergames stimulate selected parameters of skill-related physical fitness, and studies have shown such games can be effectively used for older people as a prevention from physical and cognitive decline [45,48]. One of the RCTs demonstrated the effectiveness of VR games in reference to the emotional and mental health domains measured by Short-Form Health Survey Quality of Life (SF-36). The authors proved that VR interventions can be used to improve mental health in older women with depression.

There is little research using the idea of a therapeutic garden to treat people with symptoms of depression. In 2012, in the field of positive technology, Baños et al. proposed two virtual environments that simulate environments in nature [49]. The first aimed to induce joy, and the second relaxation in elderly people. Both included scenes feature a blue sky and a greenfield through which the participant could move around. During the stroll, the voice instructed the participant and told stories to evoke specific emotions. The study concluded, that after using virtual environments, results indicated significant decreases in negative mood scores (sadness and anxiety) [49]. Nevertheless, this study used pre-post analysis from two treatment sessions, with no control group and a touch screen as a VR medium. This makes it difficult to directly relate our results to this study.

Recently, the study protocol of “Secret Garden” has appeared, which adapts the ideas of a therapeutic garden to VR goggles in order to improve the mood during the COVID-19 pandemic [50]. Unfortunately, we will have to wait for the results of this study, although a preprint of an adult study has recently appeared [51]. The study was designed as a pragmatic pilot study with 40 participants. The intervention was a weekly self-administered at-home virtual reality-based protocol consisting of two integrated parts: a 10-min 360° VR video entitled “Secret Garden” and a series of social exercises. The obtained results indicated that the applied intervention significantly reduced the intensity of depression and stress symptoms, and the obtained results were mostly maintained in the 2-week follow-up [51]. We obtained very similar results in our research.

Moreover, our findings are in line with our previous research using the VR TierOne device. By examining the use of VR therapy in chronic obstructive pulmonary disease patients, we have shown that it can significantly reduce the severity of depression symptoms and the level of stress [41]. We obtained similar results when examining the use of VR TierOne in patients with coronary artery disease (CAD) [40]. However, in both cited studies, the reduction of the tested parameters was smaller than in this study. This could be due to the higher baseline level of severity of depressive symptoms among these patients and the use of an active comparator which was Schultz Autogenic Training.

The therapeutic method used in our study was the first attempt to implement Ericksonian Psychotherapy into the virtual environment, which makes it difficult to find a direct reference to other studies. However, other psycho-social interventions that aim to improve mental health, e.g., cognitive behavioural therapy or relaxation techniques, are successfully applied in studies using the VR environment [7]. The present study demonstrated a statistically significant decrease in depression symptoms, as well as in stress and anxiety levels after the VR therapy. Similar data were published in 2014 in studies assessing the effectiveness of a VR-based stress management programme in people with mood disorders. VR-based relaxation practice using head-mounted displays significantly lowered subjective stress (*p* < 0.001), depression (*p* < 0.001) and anxiety (*p* < 0.001), and increased skin temperature (*p* < 0.001) and perceived relaxation (*p* < 0.001) in the pre-post measurement. The therapeutic intervention was based on the Neuman System Model, which provided a theoretical framework to guide this study [52]. Unfortunately, there was no control group and no patients aged over 60 in the studied group.

Important results can also be found in the paper by Navarro-Haro et al. published in 2019, since elements of mindfulness training were also used in our project. The authors found that in patients with generalised anxiety disorders, mindfulness-based virtual interventions can be as effective as a standard mindfulness procedure, but VR is a good tool to increase treatment adherence and motivation. In the cited study, the average HADS Anxiety score decreased by almost three points in the VR group, from 13.0 (SD 4.1) to 10.2 (SD 4.72) [53]. In our study, there was a similar reduction in anxiety symptoms by around three points, from 10.2 (SD 3.8) to 7.4 (SD 2.3). With regard to HADS Depression results, Navarro-Haro et al. reported reductions in the depressive mood by around two points, from 8.6 (SD 3.8) to 6.5 (SD 3.9) [53]. In our study, we observed a similar tendency in the HADS Depression results, namely a decrease from 7.1 (SD 2.0) to 5.6 (SD 2.3). However, it is worth mentioning that the average age of the patients in the study by Navarro-Haro et al. was 44.3 (SD 10.2), whereas in our study it was 70.7 (SD 4.6), thus the mood improvement is better described by the differences in the GDS, which was 12.3 (SD 4.5) in the pre-intervention versus 8.3 (SD 3.6) in the post-intervention, and 7.3 (SD 2.6) in the follow-up. This is a critical result when we take into account that these were individuals whose previous three-month multimodal therapeutic programme did not achieve the expected results.

The study above was not devoid of limitations. Firstly, due to the innovative character of the research, the current study was based on a modest sample of 23 female participants. Moreover, the applied two-week follow-up period is also a limitation of this study. The study included only individuals with depressive symptoms based on the GDS results, but without a major depressive disorder diagnosis. Thus, it is highly recommended to repeat the study with a larger group of older people taking into account a psychiatric diagnosis and observation and comparing the efficacy of VR therapy with an active comparator (e.g., individual psychotherapy). It is known that there is a high degree of placebo response in trials of psychotherapy and pharmacotherapy for depression. Secondly, the PSQ used as a secondary outcome measure, despite its high repeatability and validity, is not frequently used in scientific research. Thirdly, the scientific method used in the study is mainly based on total immersion, so if a person being examined is not, for any reason, fully “immersed” in the therapy, the effects may differ. Therefore, it would also be worth using better known diagnostic tools (e.g., Patient Health Questionnaire) and implementing a more objective way of measuring the stress level (e.g., cortisol level test), whilst assessing the participants’ real perception of the virtual world (e.g., Spatial Presence Experience Scale). We did not directly assess acceptability and feasibility; however, this form of therapy was well tolerated by participants. Future research should assess this matter quantitatively and qualitatively. Finally, the intervention group underwent an additional form of therapy, resulting in a difference in the total intervention time, which in turn could have had an impact on the results obtained. Therefore, in future research, it is worth considering comparing VR TierOne with an active comparator group.

## 5. Conclusions

Despite the aforementioned limitations, our study demonstrated that immersive virtual therapy using the idea of imagery guidance through a therapeutic garden based on the Ericksonian psychotherapy approach may significantly lower the intensity of depression symptoms, as well as the levels of anxiety and stress among older women. The previous virtual reality studies did not use techniques based on Ericksonian psychotherapy; thus, the presented results seem to be an important addendum to the current knowledge concerning the possibility of using VR therapy in older patients. However, for the same reasons, further studies on a larger sample, including men, are needed.

## Figures and Tables

**Table 1:** Participants baseline characteristic, mean (SD).

Variable	Overall (*n* = 23)	VR (*n* = 11)	Control (*n* = 12)	*p*-Value
Age, years	70.73 (4.56)	70.18 (4.87)	71.25 (4.41)	0.59 ^a^
Body mass, kg	73.14 (13.68)	74.11 (10.46)	72.25 (16.53)	0.75 ^a^
Height, cm	160.04 (6.95)	160.91 (8.44)	159.25 (5.51)	0.58 ^a^
BMI, kg/cm^2^	28.47 (4.50)	28.67 (3.74)	28.29 (5.26)	0.59 ^a^
Normal (BMI 18.5–24.9), n (%)	6 (26.09)	2 (18.18)	4 (33.33)	0.41 ^b^
Overweight (BMI 25–29.9), n (%)	9 (39.13)	5 (45.45)	4 (33.33)	0.55 ^b^
Obese (BMI > 30), n (%)	8 (34.78)	4 (36.36)	4 (33.33)	0.88 ^b^
Total cholesterol, mg/dL	196.91 (42.78)	201.18 (46.71)	193.00 (40.52)	0.66 ^a^
HDL-C, mg/dL	68.82 (16.85)	67.27 (19.02)	70.25 (15.30)	0.68 ^a^
LDL-C, mg/dL	118.78 (33.46)	125.90 (35.56)	112.25 (31.50)	0.34 ^a^
Triglycerides, mg/dL	116.00 (48.18)	114.90 (36.46)	117.00 (58.59)	0.92 ^a^
Fasting glucose, mg/dL	103.61 (13.12)	105.36 (16.56)	102.01 (9.44)	0.55 ^a^
Resting SBP, mm Hg	131.82 (13.87)	133.27 (14.99)	130.50 (13.28)	0.64 ^a^
Resting DBP, mm Hg	74.86 (8.20)	74.45 (8.10)	75.25 (8.63)	0.82 ^a^
GDS	12.26 (4.39)	12.27 (4.45)	12.25 (4.53)	0.99 ^a^
Current smoking, *n* (%)	2 (8.69)	1 (9.09)	1 (8.33)	0.95 ^b^
Back pain, *n* (%)	18 (78.26)	8 (72.72)	10 (83.33)	0.54 ^b^
Joints pain, *n* (%)	21 (91.30)	10 (90.90)	11 (91.66)	0.95 ^b^
Other pain, *n* (%)	13 (56.52)	6 (54.54)	7 (58.33)	0.85 ^b^
Marital status, married/single/widow (%)	11/3/9	5/2/4	6/1/5	0.78 ^b^
Education, low/medium/high (%)	5/10/8	3/4/4	2/6/4	0.76 ^b^

BMI: body mass index; HDL-C: high-density lipoprotein cholesterol; LDL-C: low-density lipoprotein cholesterol; ^a^ *t* test; ^b^ Chi-square test.

**Table 2:** Primary and secondary outcome measures results, mean (SD).

	VR				Control			
	Pre-Intervention (*n* = 11)	Post-Intervention (*n* = 11)	After 2 Weeks (*n* = 11)	*p*-Value	Pre-Intervention (*n* = 12)	Post-Intervention (*n* = 12)	After 2-Weeks (*n* = 12)	*p*-Value
GDS	12.27 (4.45)	8.27 * (3.60)	7.27 * (2.57)	<0.001	12.25 (4.53)	12.75 (4.82)	11.83 (2.62)	0.61
Stress level	61.45 (8.94)	52.27 * (9.08)	46.27 * (10.62)	<0.001	62.50 (9.53)	63.50 (10.37)	64.75 (11.46)	0.38
Emotional tension	21.90 (4.67)	20.09 (5.52)	17.45 * (5.75)	0.006	20.75 (2.70)	21.25 (3.91)	21.91 (4.27)	0.36
External stress	19.36 (4.90)	14.09 * (5.35	13.18 * (5.99)	<0.001	20.66 (4.29)	20.91 (3.72)	21.16 (4.74)	0.74
Intrapsychic stress	20.18 (3.81)	18.09 (2.11)	15.63 * (3.23)	0.003	21.08 (5.50)	21.33 (4.18)	21.66 (4.35)	0.77
HADS	17.27 (5.36)	13.00 * (3.25)	11.27 * (3.13)	<0.001	17.66 (4.83)	18.16 (4.52)	18.00 (3.74)	0.82
HADS-Anxiety	10.18 (3.78)	7.36 * (2.33)	5.81 * (2.60)	<0.001	9.83 (2.97)	10.00 (2.69)	10.41 (2.84)	0.48
HADS-Depression	7.09 (2.02)	5.63 (2.29)	5.45 (2.33)	0.07	7.83 (2.32)	8.16 (2.51)	7.58 (2.06)	0.55

GDS: Geriatric Depression Scale; HADS: Hospital Anxiety and Depression Scale; *p*-value as a result of Repeated Measures ANOVA; * statistically significant compared to the pre-intervention test according to Bonferroni post-hoc test.

**Table 3:** VR therapy effects, mean differences (95% CI).

	VR (*n* = 11)	Control (*n* = 12)	MS	F	*p*	ƞp^2^
GDS						
Pre vs. post	4.00 (1.11–6.89); *p* = 0.001	−0.50 (−3.27–2.27); *p* = 0.73				
Pre vs. follow-up	5.00 (2.11–7.89); *p* < 0.001	0.42 (−2.35–3.19); *p* = 0.41				
Time * group			39.47	8.31	<0.001	0.28
Stress level						
Pre vs. post	9.18 (2.65–15.71); *p* = 0.001	−1.00 (−7.25–5.25); *p* = 0.59				
Pre vs. follow-up	15.18 (8.65–21.71); *p* < 0.001	−2.25 (−8.50–4.00); *p* = 0.33				
Time * group			440.10	18.18	<0.001	0.46
Emotional tension						
Pre vs. post	1.82 (−1.42–5.06); *p* = 0.99	−0.50 (−3.60–2.60); *p* = 0.94				
Pre vs. follow-up	4.45 (1.21–7.70); *p* = 0.001	−1.17 (−4.27–1.94); *p* = 0.92				
Time * group			45.80	7.67	0.001	0.27
External stress						
Pre vs. post	5.27 (2.16–8.39); *p* < 0.001	−0.25 (−3.23–2.73); *p* = 0.98				
Pre vs. follow-up	6.18 (3.07–9.30); *p* < 0.001	−0.50 (−3.48–2.48); *p* = 0.87				
Time * group			73.17	13.28	<0.001	0.39
Intrapsychic stress						
Pre vs. post	2.09 (−0.98–5.17); *p* = 0.60	−0.25 (−3.19–2.69); *p* = 0.78				
Pre vs. follow-up	4.55 (1.47–7.62); *p* < 0.001	−0.58 (−3.53–2.36); *p* = 0.54				
Time * group			37.84	7.05	0.002	0.25
HADS						
Pre vs. post	4.27 (1.16–7.39); *p* = 0.001	−0.50 (−3.48–2.48); *p* = 0.32				
Pre vs. follow-up	6.00 (2.89–9.11); *p* < 0.001	−0.33 (−3.31–2.65); *p* = 0.75				
Time * group			62.49	11.35	<0.001	0.35
HADS-Anxiety						
Pre vs. post	2.82 (0.64–5.00); *p* = 0.003	−0.17 (−2.26–1.92); *p* = 0.79				
Pre vs. follow-up	4.36 (2.18–6.55); *p* < 0.001	−0.58 (−2.67–1.51); *p* = 0.54				
Time * group			35.61	13.18	<0.001	0.39
HADS-Depression						
Pre vs. post	1.45 (−0.32–3.23); *p* = 0.22	−0.33 (−2.03–1.36); *p* = 0.87				
Pre vs. follow-up	1.64 (−0.18–3.41); *p* = 0.10	0.25 (−1.45–1.95); *p* = 0.85				
Time * group			5.05	2.83	0.070	0.12

GDS: Geriatric Depression Scale; HADS: Hospital Anxiety and Depression Scale; *p*-value as a result of Repeated Measures ANOVA; MS: mean square; *: It is a sign of the interaction of time and group. This is the standard way of reporting the results of statistical analysis

## Data Availability

Data is available from the corresponding author on a reasonable request.
